# Efficient Prediction of Stability Boundaries in Milling Considering the Variation of Tool Features and Workpiece Materials

**DOI:** 10.3390/s23218954

**Published:** 2023-11-03

**Authors:** Huijuan Sun, Huiling Ding, Congying Deng, Kaixiang Xiong

**Affiliations:** 1 School of Mechanical Engineering and Automation, Chongqing Industry Polytechnic College, Chongqing 401120, China; sunhj@cqipc.edu.cn; 2 School of Advanced Manufacturing Engineering, Chongqing University of Posts and Telecommunications, Chongqing 400065, China; huiling_d@163.com (H.D.); scdx2013@126.com (K.X.)

**Keywords:** theoretical stability analysis, milling stability predictions, transfer learning framework

## Abstract

Theoretical stability analysis is a significant approach to predicting chatter-free machining parameters. Accurate milling stability predictions highly depend on the dynamic properties of the process system. Therefore, variations in tool and workpiece attributes will require repeated and time-consuming experiments or simulations to update the tool tip dynamics and cutting force coefficients. Considering this problem, this paper proposes a transfer learning framework to efficiently predict the milling stabilities for different tool–workpiece assemblies through reducing the experiments or simulations. First, a source tool is selected to obtain the tool tip frequency response functions (FRFs) under different overhang lengths through impact tests and milling experiments on different workpiece materials conducted to identify the related cutting force coefficients. Then, theoretical milling stability analyses are developed to obtain sufficient source data to pre-train a multi-layer perceptron (MLP) for predicting the limiting axial cutting depth (*a_plim_*). For a new tool, the number of overhang lengths and workpiece materials are reduced to design and perform fewer experiments. Then, insufficient stability limits are predicted and further utilized to fine-tune the pre-trained MLP. Finally, a new regression model to predict the *a_plim_* values is obtained for target tool–workpiece assemblies. A detailed case study is developed on different tool–workpiece assemblies, and the experimental results validate that the proposed approach requires fewer training samples for obtaining an acceptable prediction accuracy compared with other previously proposed methods.

## 1. Introduction

Continuous developments in the manufacturing industry have pushed the machine tool to pursue a higher machining efficiency and accuracy. However, chatter vibrations occurring in the milling process will restrict the machine tool to its limits [1]. Since regenerative chatter is the most common chatter phenomenon in milling, in-depth methods have been developed to suppress the chatter occurrences [2,3]. Providing accurate stability lobe diagrams (SLDs) has become a major research direction to select chatter-free machining parameters. The SLDs describe the relationships between the spindle speed and axial cutting depth, which are derived by solving the dynamic model of the tool and workpiece system [4]. Altintas and Budak [4,5] developed a two-degree-of-freedom (2DOF) analytical milling chatter model and provided a Fourier-approximation-based method to obtain the chatter-free machining parameters in the frequency domain. Since this method was proven to be effective in determining the SLD, it lays a foundation for predicting the chatter stability. Subsequently, the semi-discretization method, full-discretization method and time-domain method were also put forwarded to construct the chatter vibration models and conduct the machining stability analyses [6]. The groundbreaking work of researchers is beneficial for operators to correctly select the processing parameters. Among these chatter vibration models, the tool tip dynamics and cutting force coefficients are the critical inputs of the dynamic model.

The tool tip dynamics can reflect the structural dynamics characteristics of the machining system and are commonly represented by the tool tip frequency response functions (FRFs) in the frequency domain [7]. Generally, the tool tip FRFs are acquired through impact testing, finite element simulations, substructure coupling methods and so on [8,9,10,11]. Kolar et al. [12] proposed a coupled method to establish the whole machine tool system by joining the spindle and machine frame finite element (FE) model. It is convenient to establish the FE model of a new tool; however, its related joint dynamics are difficult to simulate accurately. Due to some simplifications in constructing the finite element model, the frequency response function obtained by the finite element simulations still have some obvious deviations from the actual values. Wang [13] introduced the receptance coupling substructure analysis (RCSA) method to utilize the tested FRFs at the end of the spindle to predict the tip FRFs of different tool–holder assemblies. However, the accuracy of RCSA is subject to many parameters that are difficult to measure or identify. For example, the joint dynamics of the tool–holder–spindle assembly are difficult to measure and susceptible to the complex contact conditions, limiting the ability to accurately identify the joint stiffness and damping coefficients. Therefore, impact testing has become the most acceptable approach to obtain the tool tip dynamics; this is where the forces exerted at the tool tip are measured and combined with the vibration signals recorded by the pasted sensors to directly generate the tool tip FRFs. Chang et al. [14] utilized impact testing to obtain the FRFs of the tool–spindle system and predicted the SLD using the full discretization method for selecting the appropriate chatter-free machining parameters. Since the tool tip FRFs are mainly determined by the dynamic characteristics of the tool–holder assembly, the tool properties, including the tool materials, tool diameter and tool overhang length, can significantly affect the tool tip FRFs. Once one of the mentioned tool properties is changed, repeated experiments, simulations or identifications are required to update the tool tip FRFs, and the shutdown of the machine tool for a long time will decrease the production efficiency. Therefore, though many effective methods have been proposed to improve the accuracy of tool tip FRFs, their efficient evaluation under different tool–holder assemblies remains a challenge.

Besides the tool tip dynamics, the machining stability is also seriously affected by the cutting force coefficients. Generally, the experimental data obtained from the milling tests under different combinations of machining parameters are used to evaluate the cutting force coefficients through the orthogonal-to-oblique transformation and average calibration method [15,16]. Experimental results show that the workpiece material can affect the cutting force coefficients. Lacerda et al. [17] carried out milling tests on GH-190 cast iron and ABNT 1020 steel and calculated their cutting force coefficients, and the SLDs were plotted and compared to confirm that the milling stability was affected by the workpiece-material-dependent cutting force coefficient. Yu et al. [18] carried out a series of single-factor milling tests on TiB2/Al composites and other three aluminum alloys to compare their cutting performances; the cutting force coefficients of each material were identified and used to provide theoretical guidance for selecting reasonable milling parameters for the TiB2/Al composites. Qiu [19] et al. took four workpieces with the materials TC4, 7075T6, 45 steel and 304 stainless steel, respectively, for a case study and validated that the variation of the workpiece material had a great impact on the milling stability through changing the cutting force coefficients. Currently, the milling stability prediction models are mainly established for one workpiece material, and poor accuracies will be obtained when directly using them to predict the milling stability of a new material. Therefore, when facing different workpiece materials, repeated milling tests are required to update the cutting force coefficients and establish a new milling stability prediction model, which is extremely time-consuming. Some researchers have already focused on predicting the surface roughness for different workpiece materials, but an efficient prediction of milling stability under multiple workpiece materials has been rarely discussed [20].

According to the above descriptions, properties of the tool-workpiece will have a comprehensive impact on the SLDs, and only using the impact testing and milling tests to realize the milling stability prediction under multiple machining conditions can be costly and inefficient. Then, current research is using fewer required experimental data to efficiently establish a milling stability prediction model considering multiple influencing factors, such as the tool material, tool diameter, tool overhang length and workpiece material. Considering this situation, transfer learning aiming to apply the knowledge learned in the source domain to solve similar problems in the target domain has been introduced to benefit the milling stability analysis [21]. Unver et al. [22] proposed a new transfer learning (TL) framework for chatter detection based on the deep learning and numerical chatter simulation. Only an impact hammer test was required to generate different IMFs close to the modal frequencies for training different AlexNets. Predictions of these AlexNets were ensembled to output the vibration state, and they were directly transferred to the actual working condition to perform the chatter detection. This method indicates a significant potential in avoiding expensive experimental data collection, but it has not further taken the change of tool and workpiece material into consideration. Liu et al. [23] constructed the source domain dataset by sufficient impact tests on the source tool and divided the sub-workspace based on the modal order of FRF, and only a small number of impact tests were performed in each sub-workspace on the target tool to construct the target data. For each sub-workspace, the source data and target data were combined to train a modal parameters prediction model of the target tool through the transfer learning algorithm TrAdaBoost.R2. To further study the tool-dependent milling stability, Deng et al. [24] developed the method proposed by Liu et al. for efficiently predicting the SLDs for different tool–holder assemblies. A source tool was selected to perform the impact tests at sufficient overhang lengths to construct the source milling stability data, and the impact tests only at three tool overhang lengths were required to construct the target data for training the overhang length-dependent milling stability prediction model. Moreover, Postel et al. [25] further considered the differences between the theoretical and experimental stability limits and utilized fewer experimental data to fine-tune the tool overhang length-dependent deep neural network pre-trained using sufficient theoretical stability data. However, this research only focused on the stability states and could not further deal with the efficient SLD predictions with various tool–workpiece assemblies. The introduction of different effective transfer learning methods benefited evaluating the milling stability with less required data. Nevertheless, the stability prediction models in most studies were still limited to one specific tool or one specific workpiece material, which were not applicable to the multiple machining conditions with various tool features and workpiece materials.

Considering the diversity of tools and workpieces in machining processes and aiming to reduce the cost of training a milling stability model, this paper presents an approach to efficiently predicting the tool–workpiece assembly dependent milling stability. This can be achieved by utilizing the transfer learning for a neural network. First, a source tool was selected to carry out the impact tests to obtain the tool tip FRFs for multiple overhang lengths, and milling tests were conducted on different workpiece materials to identify related cutting force coefficients. These evaluated parameters were used to predict the limiting axial cutting depths and construct the source data through the theoretical milling stability analysis. Then, a multilayer perceptron was trained using the source data to predict the *a_plim_* values under multiple milling conditions. For a new tool, fewer overhang lengths and workpiece materials were selected to conduct the impact tests and milling tests for constructing the target stability data. The pre-trained network was then fine-tuned with the target data for more accurate stability predictions under the target milling conditions. The main goal of this presented approach is to keep the measurement effort to a minimum, making it a promising approach to performing an efficient stability prediction of the machining process with diverse tools and workpieces.

The remainder of this paper is organized as follows: In Section 2, the necessity for considering the properties of the tool-workpiece in the milling stability prediction is discussed, and a framework to establish the stability prediction model for different tool–workpiece assemblies is also provided. A detailed case study is carried out in Section 3 to explain the application of the proposed approach and provide an experimental validation. The work is summarized in Section 4.

## 2. Materials and Methods

### 2.1. Theoretical Analysis of Milling Chatter Stability

**Figure 1:**
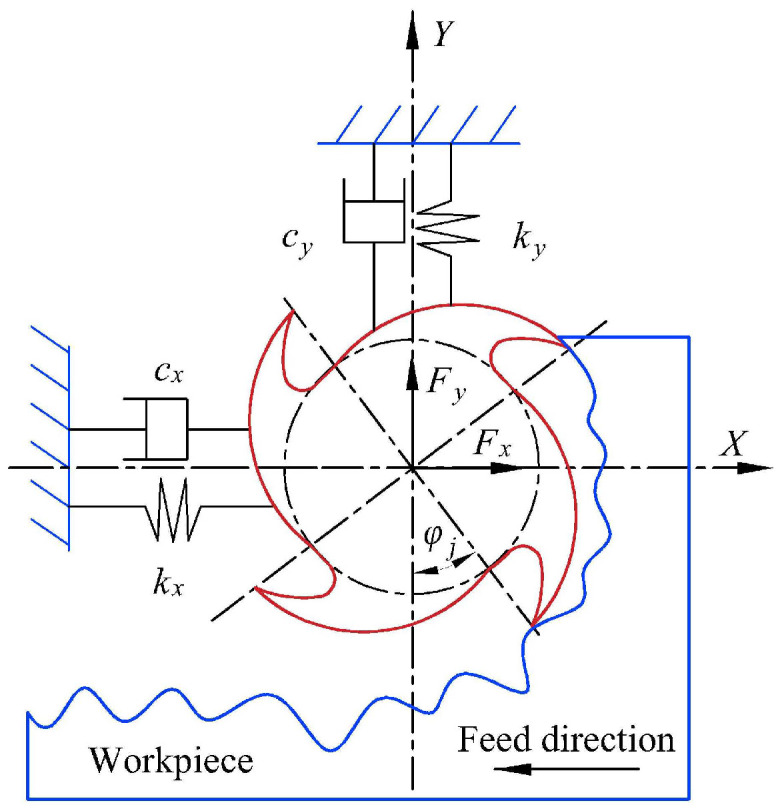
Dynamic models of milling process with two degrees of freedom.

The dynamic model of the milling process is the basis for developing the milling stability analysis. By solving the milling dynamic model, the limiting axial cutting depth can be obtained, and then the SLDs can be plotted within the spindle speed range. Generally, the milling process is described as a dynamic model with two degrees of freedom (2-DOF) in the X and Y directions as shown in Figure 1.

With this 2-DOF model, Altintas and Budak [4] proposed a zero-order approximation (ZOA) method, and it has been widely used to conduct the milling stability analysis. According to the ZOA method, the limiting axial cutting depth *a_plim_* and related spindle speed can be calculated using Equation (1):

(1) $$\begin{array}{l} a_{plim}=-\frac{2\pi \Lambda_{R}\left[1+{\left(\Lambda_{I}/\Lambda_{R}\right)}^{2}\right]}{NK_{tc}}\\ n=\frac{60\omega_{c}}{\pi N(2k+1)-2\arctan(\Lambda_{I}/\Lambda_{R})} \end{array}$$

Here, *N* is the tool teeth number, *K_tc_* is the tangential force coefficient, *ω_c_* is the chatter frequency, *k* is the lobe number, and *Λ_R_* and *Λ_I_* are the real and imaginary parts of the system’s eigenvalue in Equation (2), respectively.

(2) $$\begin{array}{l} \Lambda =\left(\Lambda_{R}+\Lambda_{I}i\right)=-\frac{1}{2a_{0}}\left(a_{1}\pm \sqrt{a_{1}{}^{2}-4a_{0}}\right)\\ a_{0}=G_{xx}G_{yy}\left(\alpha_{xx}\alpha_{yy}-\alpha_{xy}\alpha_{yx}\right)\\ a_{1}=\alpha_{xx}G_{xx}+\alpha_{yy}G_{yy} \end{array}$$

Here, *G_xx_* and *G_yy_* are the tool tip FRFs in the *x* and *y* directions, respectively, and *α_xx_*, *α_xy_*, *α_yx_* and *α_yy_* are the direction coefficients:

(3) $$\begin{array}{l} G_{xx(yy)}=\sum_{r=1}^{n}\frac{\omega_{r}^{2}/k_{r}}{\omega_{r}^{2}-\omega^{2}+i2\xi_{r}\omega \omega_{r}}\\ \alpha_{xx}=\frac{1}{2}{\left[\cos2\phi -2K_{rc}\phi +K_{rc}\sin2\phi \right]}_{\phi_{st}}^{\phi_{ex}}\\ \alpha_{xy}=\frac{1}{2}{\left[-\sin2\phi -\phi +K_{rc}\cos2\phi \right]}_{\phi_{st}}^{\phi_{ex}}\\ \alpha_{yx}=\frac{1}{2}{\left[-\sin2\phi +2\phi +K_{rc}\cos2\phi \right]}_{\phi_{st}}^{\phi_{ex}}\\ \alpha_{yy}=\frac{1}{2}{\left[-\cos2\phi -2K_{rc}\phi -K_{rc}\sin2\phi \right]}_{\phi_{st}}^{\phi_{ex}} \end{array}$$

Here, *ω_r_*, *k_r_* and *ξ_r_* are the modal frequency, modal stiffness and modal damping ratios; *k_rc_* is the ratio of the radial and tangential cutting force coefficients and *ϕ*_st_ and *ϕ*_ex_ are the start and exit angles of the cutting tooth.

As can be seen from Equations (1) to (3), *a_plim_* is dependent on the tool tip FRFs and cutting force coefficients. Then, changes in the tool features such as the tool diameter and tool overhang length affect the milling stability by changing the tool tip FRFs; in addition, changes in the workpiece materials affect the milling stability by changing the cutting force coefficients. Therefore, it is necessary to develop a tool–workpiece assembly dependent milling stability prediction method.

**Figure 2:**
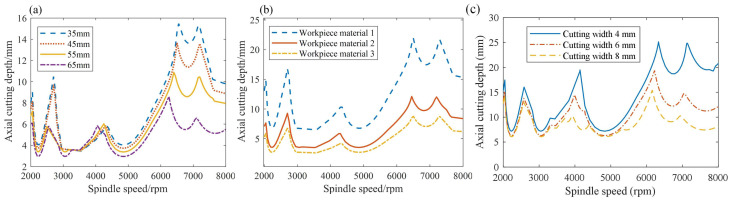
The SLDs for different tool overhang lengths, workpiece materials and cutting widths. (**a**) Different tool overhang lengths, (**b**) Different workpiece materials, (**c**) Different cutting widths.

The SLDs for different tool overhang lengths and workpiece materials are provided in Figure 2a,b, respectively. In Figure 2a, four tool overhang lengths are selected to plot the SLDs for a specific workpiece material, and most *a_plim_* values decrease with the increase in the tool overhang lengths. In Figure 2b, the SLDs are plotted for three different workpiece materials at a specific tool overhang length, and deviations in the *a_plim_* values for different SLDs can be observed. However, whether it is the changes in the tool overhang length or the workpiece materials, the variation tendencies of these lobes are similar. Therefore, these similarities may lay a foundation for the application of transfer learning in the milling stability under variable machining conditions. Moreover, the SLDs also varied with the change in the cutting width as shown in Figure 2c.

### 2.2. Specifications of the Transfer Leaning

Transfer learning can utilize the extracted useful knowledge from the source task to benefit the modeling of the target domain, which requires fewer target data and accelerates the modeling process. Transfer learning is usually divided into four types: the instance-based transfer learning, feature-based transfer learning, model-based transfer learning and relation-based transfer learning [26,27,28]. The model-based transfer learning emphasizes that the model parameters and weights obtained from similar tasks or previous experiences can be used to improve the performance of the machine learning model on a small number of target samples. This meets the requirements for predicting milling stability under different tool–workpiece assemblies, as the parameters and weights of a source tool stability model can be transferred to assist in the stability modeling of a target tool with fewer target data. According to the concept and term of transfer learning, some symbols and definitions for the model-based transfer learning are organized as follows:

The domain of transfer learning contains various labeled or unlabeled data, which are represented as domain data *D* = {*X*, *Y*}, where *X* is the instance space and *Y* is the label space. The source domain *D_s_* contains the existing knowledge, and the target domain *D_t_* is the domain to be learned. In general, the sample sizes of the source domain and the target domain are different: the source domain contains sufficient data required to complete the task *T*, and the target domain has only a small number of representative samples.

The (*i*-th) instance *x_i_* of *X* is presented by the feature vector, and its corresponding label is defined as *y*_i_. For m-many samples, the domain data can be described as *D* = {(*x*_1_, *y*_1_), (*x*_2_, *y*_2_),…, (*x*_i_, *y*_i_),…, (*x*_m_, *y*_m_)}, where *x_i_* ∈ *X*, *y_i_* ∈ *Y*, *i* = 1, 2, …, *m*. {*X_s_*, *Y_s_*} represent the instance and label spaces, respectively, for the source domain data *Ds*, and {*X_t_*, *Y_t_*} represents the instance and label spaces, respectively, for the target domain data *D_t_*.

*f*(·) denotes a prediction function describing the mathematic relationship between the instance space and label space, and it can be accurately obtained with sufficient training samples. *f_s_*(·) and *f_t_*(·) are the prediction models for the source and target domains, respectively.

Task T is defined by the label space *Y* and prediction model *f*(·). *T* = {*Y*, *f*(·)}learns from the feature vector and label space {*x_i_*, *y_i_*} to obtain a prediction function *f*(·). When giving the source domain *D_s_* and target domain *D_t_*, training *f_s_*(·) using the source domain data is the source task *T_s_*. With the help of *f_s_*(·), the target task *T_t_* is to finetune the *f_s_*(·) through the target domain data to obtain the *f_t_* (·).

### 2.3. Tool and Workpiece-Dependent Milling Stability Prediction Based on Transfer Learning

Currently, machine learning has been widely used to predict the stability of the milling process considering various influencing factors [29]. Generally, sufficient training data are required to train a machine learning model and guarantee its accuracy. When facing the various properties of different tool–workpiece assemblies, a lot of experiments are required to obtain sufficient data, and the costs of the model training may be sharply increased. Therefore, transfer learning is introduced in this paper to benefit the milling stability model with fewer training samples. To realize model-based transfer learning, obtaining an accurate pretrained prediction model and selecting an appropriate finetuning strategy are two important foundations.

#### 2.3.1. Pretraining of a Milling Stability Prediction Model

**Figure 3:**
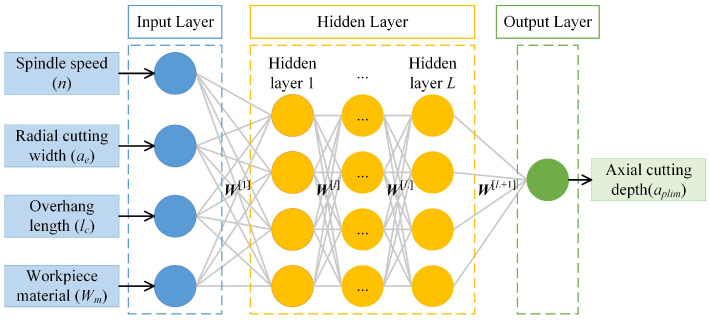
The topological structure of an MLP.

The artificial neural network has been widely used in predicting the milling stability and can achieve an acceptable prediction accuracy [30,31]. Then, the multilayer perceptron is used to predict the limiting axial cutting depth. The MLP is a feedforward artificial neural network consisting of the input layer, hidden layers, and the output layer. The topological structure of an MLP containing *L* hidden layers is shown in Figure 3, where *W*^[*l*]^ is the weight vector connecting the (*i* − 1)th and (*l*-th) layers, *l* = 1, 2,…, *L* + 1. Each layer has one or more neurons, and the neurons in two adjacent layers are fully connected. Then, the input of the (*j*-th) neuron in the *l*th layer is the weighted sum of the outputs of all neurons in the (*i* − 1)th layer.

(4) $$neuron_{i,j}=f_{n}\left(\sum_{j}neuron_{i-1,j}\times w_{j}+b_{j}\right)$$

Here, *w_ij_* is the weight connecting the (i-th) neuron in the (*i* − 1)th layer and the (*j*-th) neuron in the *l*th layer, *b_j_* is the bias of the (*j*-th) neuron in the (*l*-th) layer, and *f_n_*() is the activation function of the *(j*-th) neuron in the (*l*-th) layer. The commonly used activation functions include the ReLU, sigmoid, tanh and softmax.

Before training an MLP neural network, the number of hidden layers and the number of neurons in each layer are first defined. The number of neurons in the input layer and output layers is equal to the number of elements in the input and output vectors, respectively. Then, the weights and biases are randomly initialized and further modified using the Stochastic Gradient Descent (SGD) in Equation (5) [32].

(5) $$\theta =\theta -\eta \cdot \nabla_{\theta }J(\theta ;x^{\left(i\right)};y^{\left(i\right)})$$

Here, *η* is the learning rate, *J*() is the loss function and (*x*^(*i*)^, *y*^(*i*)^) is the training set data pair.

According to the theoretical analysis of milling stability, the limiting axial cutting depth depends on the tool features, workpiece materials and machining parameters; then, the input vector of the milling stability prediction model should contain these factors. The tool features and machining parameters have already been considered to construct the milling stability prediction model, but they are still limited to one specific workpiece material. To reduce the number of training samples and improve the generalization performance of the trained stability prediction model under multi-milling conditions, the machining parameters, tool features and workpiece materials are combined to be the input variables *x* = {*n*, *a_e_*, *l_c_*, *W_m_*} of an MLP to predict the limiting axial cutting depth *a_plim_*. The terms *n*, *a_e_*, *l_c_* and *W_m_* represent the spindle speed, radial cutting width, tool overhang length and workpiece material, respectively. Moreover, one-hot encoding is adopted to express the workpiece material since it cannot be directly evaluated using a numerical value. One-hot encoding uses the *P*-bit register to code the *P* states of the feature, ensuring that each state has an independent register bit and only one of them is valid at any time. When encoding the (*i*-th) workpiece material, the (*i*-th) bit of the register is marked as 1, and the remaining bits are marked as 0. On this basis, a source MLP for predicting the milling stability can be constructed using sufficient training samples.

#### 2.3.2. Finetuning for Training a Milling Stability Model on the Target Domain

After the pretraining model is constructed, it is necessary to select one appropriate finetuning strategy to retrain the model parameters. Global finetuning and local finetuning are two commonly used finetuning strategies.

Global finetuning retrains all the weights of the pretrained model, and local finetuning first freezes some layers of the pretrained model and updates the weights in the remaining layers. Since global finetuning can affect all the parameters of the whole model when fine-tuning, a better adaptation to the target dataset is achieved. However, its convergence speed is higher than that of local finetuning. Local finetuning often freezes the parameters of the first layers and finetunes the parameters of the last layers, for the first layers usually learn more basic low-level features and the learned features will be more abstract and advanced as the number of network layers increases. However, using the local finetuning for a pretrained model with fewer layers, only fewer parameters can be retrained after some layers are frozen, and then the performance of the neural network may be greatly affected by the source domain dataset [33]. Thus, the finetuning strategy can be determined according to the structure of the source milling stability prediction model.

Then, transfer learning in efficiently predicting the tool and workpiece-dependent milling stability can be summarized as follows:

A source tool is first selected to construct the source data *D_s_* = {*X_s_*, *Y_s_*} shown in Equation (6). The instance space *X_s_* is composed of *m_s_* feature vectors, the (*i*-th) of which can be defined as *x_si_* = {*n_si_*, *a_esi_*, *l_csi_*, *W_msi_*}, *i* = 1, 2, …, *m_s_*. The label space *Y_s_* is composed of *m_s_ a_plim_* values, where *y_si_* = {*a_plimsi_*}, *i* = 1, 2, …, *m_s_*.

(6) $$D_{s}=\left\{\left(x_{si},y_{si}\right)\right\},\mathrm{where}x_{si}\in X_{s},y_{si}\in Y_{s},\mathrm{when}i=1,2,\ldots ,m_{s}$$

For a new tool, the target data *D_t_* = {*X_t_*, *Y_t_*} are described in Equation (7). Similarly, the instance space *X_t_* is composed of mt feature vectors, the (*i*-th) of which can be defined as *x_ti_* = {*n_ti_*, *a_eti_*, *l_cti_*, *W_mti_*}. The label space *Y_t_* is composed of *m_t_ a_plim_* values, where *y_ti_* = {*a_plimti_*}, *i* = 1, 2, …, *m_t_*. The source data size *m_s_* is always much larger than the target data size *m_t_*.

(7) $$D_{t}=\left\{\left(x_{ti},y_{ti}\right)\right\},\mathrm{where}x_{ti}\in X_{t},y_{ti}\in Y_{t},\mathrm{when}i=1,2,\ldots ,m_{t}$$

An accurate MLP is trained using the sufficient source data *Ds*, and it is transferred to the target domain. A finetuning strategy is selected according to the structure of the source MLP. With the finetuning strategy, the corresponding weights of the source MLP are re-trained using the target data *D_t_*. On this basis, a new MLP accurately mapping the target instance *x_ti_* to its label *y_ti_* can be obtained with only fewer target samples.

**Figure 4:**
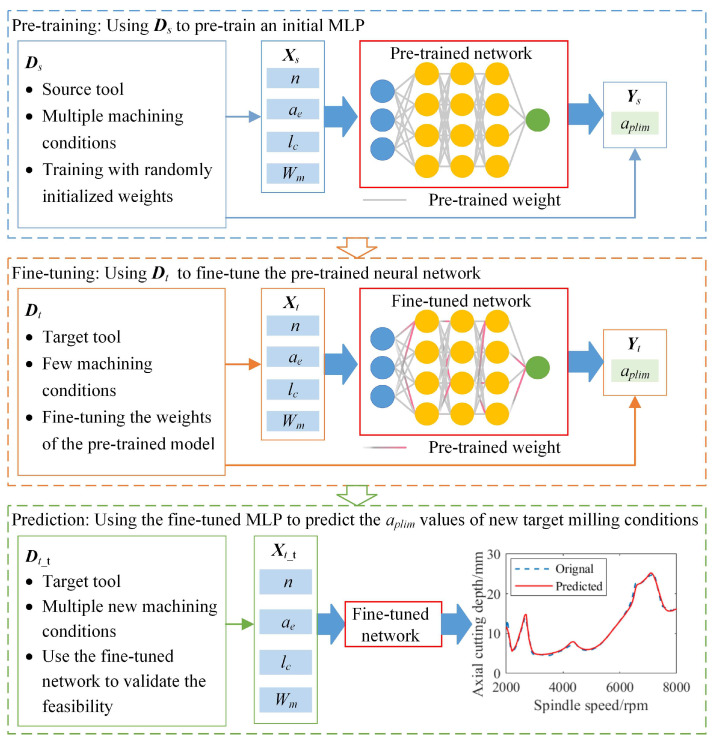
Transfer learning in predicting the milling stability.

A concise description of transfer learning in predicting the milling stability under different combinations of a tool and workpiece is provided in Figure 4. First, different overhang lengths of the source tool are selected to conduct the impact tests at the tool tip and obtain the FRFs. The workpieces with different materials are determined, and the source tool is adopted to perform several milling tests on each workpiece. Then, the cutting force coefficients for these workpieces are identified and combined with the obtained tool tip FRFs to compute the limiting axial cutting depths through the theoretical milling stability analysis. With the sufficient source data *D_s_* composed of the machining parameters, tool overhang length, codes of the workpiece materials and limiting axial cutting depths, an MLP is trained to predict the *a_plim_* values of the source tool. For the target tool, reduced tool overhang lengths and workpiece materials are defined to perform the impact tests and milling tests. The obtained tool tip FRFs and cutting force coefficients are utilized to construct the target data *D_t_*. The structural parameters of the pretrained source MLP are transferred to the target domain, and the target data are utilized to update the weights through the selected finetuning strategy. Ultimately, a new MLP is trained to predict the milling stability of the target tool for different combinations of overhang length and workpiece material.

## 3. Case Study

**Figure 5:**
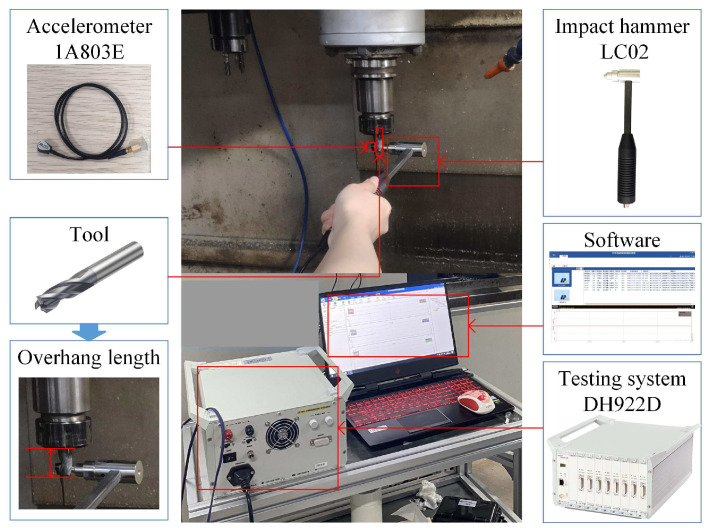
Impact testing on the machine tool and related experimental instruments.

In this section, we describe how the proposed transfer learning-based approach was used to carry out a detailed case study on a 3-axis vertical machining center, as shown in Figure 5. The experimental conditions and the data construction are introduced in Section 3.1. The details of constructing and fine-tuning the pretrained model to obtain the milling stability model of a target tool under multiple machining conditions are described in Section 3.2 and Section 3.3, and comparisons to the existing method are also provided in these sections.

### 3.1. The Experiments and Data Construction

To validate the feasibility of the proposed method in predicting the milling stability under various tool–workpiece assemblies, three tools differing in the diameter, material, teeth number and total length were selected, and each tool was designed to cut three workpieces with different materials, such as cast iron, 45 steel and aluminum 6061. With the one-hot encoding, the aluminum 6061, cast iron and 45 steel were represented by 001, 010 and 100. The specification information of the tools and workpiece materials are described in Table 1 and Table 2. The tool with a diameter of 12 mm was selected as the source tool *T*_2_, and the other two tools *T*_1_ and *T*_3_ with diameters 8 mm and 16 mm, respectively, were taken as the target tools.

For the source tool *T*_2_, sufficient stability data were required to construct an accurate stability prediction model. Then, within the variation range of the source tool overhang length [35 mm, 70 mm], eight discrete values were selected at a small interval of 5 mm to perform the impact tests at the tool tip for obtaining the corresponding FRFs in the *X* and *Y* directions as shown in Figure 5. Furthermore, the source tool was used to perform several full-immersion down-milling experiments of cast iron, 45 steel and aluminum 6061 under different *f_z_* values, and the mean cutting forces in three directions were measured and utilized to identify the corresponding radial and tangential cutting force coefficients, as listed in Table 2 through the regression analysis.

For the target tools *T*_1_ and *T*_2_, since the aim was to construct the target milling stability prediction model with fewer impact tests and milling experiments, only four discrete tool overhang lengths separated by 10 mm and two types of workpiece materials were selected for each target tool as listed in Table 3. Similarly, the target tool tip FRFs corresponding to these tool overhang lengths were obtained through the impact tests, and several full-immersion down-milling experiments under different *f_z_* values were carried out to measure the mean cutting forces and to identify the related cutting force coefficients shown in Table 2. In addition, four other discrete tool overhang lengths and one type of workpiece material for each target tool were also selected according to Table 3 to perform the corresponding impact tests and milling tests to obtain the tool tip FRFs and cutting force coefficients, which were only utilized to construct the target testing data for further validating the accuracy of the target milling stability prediction model.

These obtained tool tip FRFs and cutting force coefficients were used to conduct the theoretical milling stability analysis to construct the source and target data. For the source tool *T*_2_, the Latin hypercube sampling method was used to sample 150 combinations of *a_e_*, *l_c_* and *W_m_* within their variation ranges. For each combination of *a_e_*, *l_c_* and *W_m_*, 121 pairs of *n* and *a_plim_* were computed through Equations (1)–(3) within the spindle speed range of 2000 rpm to 8000 rpm at an interval of 50 rpm. Thus, there were 121 × 150 = 18150 combinations of *n_s_*, *a_es_*, *l_cs_*, *W_ms_* and *a_plims_* to construct the source data *D*_s_*T*2_. For each target tool, only 150 × 0.1 = 15 combinations of *a_e_*, *l_c_* and *W_m_* were selected using the Latin hypercube sampling method, where the *l_c_* had four different values and the *W_m_* had two different material types, and each combination was used to compute 61 pairs of *n* and *a_plim_* within the spindle speed range of 2000 rpm to 8000 rpm at an interval of 100 rpm. Then, there were 61 × 15 = 915 combinations of *n_t_*, *a_et_*, *l_ct_*, *W_mt_* and *a_plimt_* for each target tool, meaning that the target data *D_t_T_*_1_ and *D_t_T_*_3_ for the target tools *T*_1_ and *T*_3_ both had 915 training samples. Moreover, to construct the target testing data, the eight tool overhang lengths and three workpiece materials for each target tool were also taken to compose 150 combinations of *a_e_*, *l_c_* and *W_m_* using the Latin hypercube sampling method, and 121 pairs of *n* and *a_plim_* were computed at an interval of 50 rpm for each combination. Therefore, there were 121 × 150 = 18150 combinations of *n*, *a_e_*, *l_c_*, *W_m_* and *a_plim_* for the target testing data *D_t_T_*_1*t*_ and *D_t_T_*_3*t*_, respectively.

### 3.2. Details in Constructing a Pretraining Model for the Source Tool

Here, 80% of the source data *D_s_T_*_2_ was randomly selected to be the training data, and the remaining 20% was taken as the testing data. The mean absolute percentage error (MAPE), root mean square error (RMSE) and coefficient of determination(*R*^2^) described in Equation (8) were introduced to verify the accuracy of the trained prediction model. The MAPE reflects the deviation degree of the predicted value from the actual value, and a smaller MAPE corresponds to a higher accuracy. The RMSE is the difference between the predicted and actual values; a smaller RMSE indicates that the predicted values are close to the actual ones. *R*^2^ stands for the fitting performance of the regression model, which varies from 0 to 1, and a value of *R*^2^ closer to 1 means a better fitting degree.

(8) $$\begin{array}{l} \mathrm{MAPE}=\frac{100\%}{m}\sum_{i=1}^{m}\left(\left|y_{pi}-y_{i}\right|/y_{i}\right)\\ \mathrm{RMSE}=\sqrt{\frac{1}{m}{\sum_{i=1}^{m}\left(y_{pi}-y_{i}\right)}^{2}}\\ R^{2}=1-\frac{\sum_{i}{\left(y_{pi}-y_{i}\right)}^{2}}{\sum_{i}{\left(y_{ave}-y_{i}\right)}^{2}} \end{array}$$

Here, *m* is the total number of samples, *y_i_* and *y_pi_* represent the actual and predicted values, respectively, and *y_ave_* is the average of the real value.

The multilayer perceptron was first selected to train the regression model. The input layer had seven neurons representing *n*, *a_e_*, *l_c_* and *W_mt_*, respectively, and the output layer had one neuron representing *a_plim_*. Several MLPs with different parameters were initially trained and evaluated using the indexes listed in Equation (8), and the configurations listed in Table 4 showed a better performance. Then, they were finally determined to establish the milling stability model for the source tool *T*_2_, and the learning rate was set to 0.001. With the 18,150 × 0.8 = 14,520 training samples and the 18,150 × 0.2 = 3630 testing samples, the MLP was trained to predict the *a_plim_* values and further used to calculate the MAPE, RMSE, and *R*^2^ values, as listed in Table 5. The MAPE 0.0271, RMSE 0.331 and *R*^2^ 0.998 validated the accuracy of the constructed MLP. Furthermore, the source data *D_s_T_*_2_ were also used to train other two regressors, Decision Tree (DT) and Random Forest (RF), and the corresponding MAPE, RMSE and *R*^2^ values were also calculated as listed in Table 5. Comparing these values in Table 5, the MLP had the smallest MAPE and RMSE values and the largest *R*^2^ value, validating that the MLP better approximated the relationship between the machining conditions and the milling stability.

**Figure 6:**
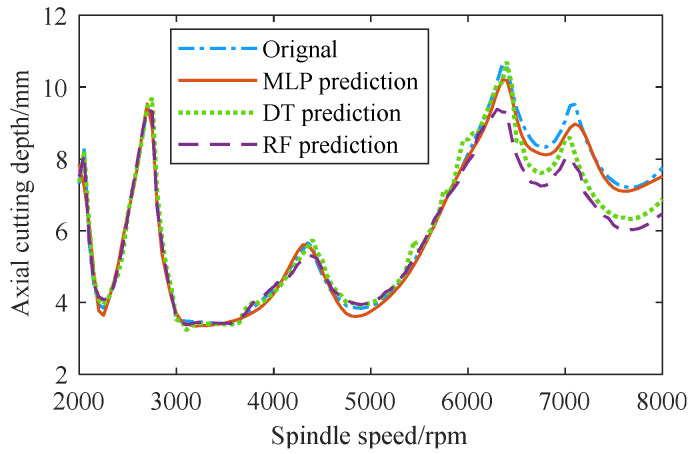
SLDs plotted by the MLP, DT and RF models.

In addition, three SLDs plotted by the MLP, DT, and RF models, respectively, were compared in Figure 6. When *n* > 6300 rpm, the *a_plim_* values predicted by the DT and RF models showed obvious deviations from the original ones, while the *a_plim_* values predicted by the MLP model still fit the original ones well. Therefore, the MLP was selected to construct the pretraining model for predicting the milling stability under the multiple machining conditions of the source tool.

In this work, the machining parameters, tool features and workpiece materials were taken as the inputs of the prediction model, which is different from the traditional prediction model only for one specific workpiece material. Therefore, the advantage of taking the workpiece material as an input when establishing the milling stability prediction model is further discussed. First, the 150 combinations of *a_e_*, *l_c_* and *W_m_* for the source tool *T*_2_ were divided into three parts according to the workpiece materials: *W_m_*_1_, *W_m_*_2_ and *W_m_*_3_. Then, each workpiece material corresponded to 50 combinations of *a_e_* and *l_c_*, which were used to compute 50 × 121 = 6050 pairs of *n_s_* and *a_plim_*. Thus, there were three different datasets, namely, *D_s_Wm_*_1_, *D_s_Wm_*_2_ and *D_s_Wm_*_3_, for the three workpiece materials. Each dataset contained 6050 combinations of *n*, *a_e_*, *l_c_*, *W_m_* and *a_plim_*, 80% of which was randomly selected as the training data and the other 20% of which was taken as the testing data. On this basis, three different MLPs, namely, MLP_*W_m_*_1_, MLP_*W_m_*_2_ and MLP_*W_m_*_3_, were established for the workpiece materials *W_m_*_1_, *W_m_*_2_ and *W_m_*_3_, respectively, to predict the corresponding *a_plim_* values. For each workpiece material, other 50 combinations of *a_e_* and *l_c_* were randomly selected to construct 50 × 121 = 6050 testing samples for validating the feasibility of the constructed MLP. Moreover, the obtained 6050 × 3 = 18,150 testing samples for three workpiece materials were also input to the previously constructed source pretraining model to predict the *a_plim_* values. With these testing samples, the calculated MAPE, RMSE and *R*^2^ values for different MLPs are listed in Table 6. Comparing these evaluation index values, the previously obtained source pretraining model showed a higher prediction accuracy than the other three MLPs on the same testing data. For instance, the MAPE values for the three testing datasets of the workpiece materials *W_m_*_1_, *W_m_*_2_ and *W_m_*_3_ were decreased by 22%, 14% and 29% when using the source pretraining model. This indicates that taking the workpiece material as the input feature can improve the generalization ability of the prediction model. Therefore, it is reasonable and effective to add the workpiece material as an input feature.

### 3.3. Transfer Learning from the Source Tool to the Target Tools

#### 3.3.1. Fine-Tuning of the Pretrained Source Model

According to the parameters shown in Table 4, the pretrained MLP in Section 3.2 was a five-layer neural network with a simple structure. Then, the global finetuning strategy was selected to develop the transfer learning. The target training data *D_t_T_*_1_ and *D_t_T_*_3_ obtained in Section 3.1 were used to finetune the pretrained MPL, respectively. The target testing data *D_t_T_*_1t_ and *D_t_T_*_3t_ obtained in Section 3.1 were taken as the testing datasets, and the calculated values of the evaluation indexes MAPE, RMSE and *R*^2^ are listed in Table 7. Moreover, to verify the feasibility of the proposed pre-training and finetuning-based milling stability prediction model, two other types of milling stability prediction models were taken for comparisons. One was the pretrained source prediction model, and the other was the regressor constructed by only using the target data.

**Figure 7:**
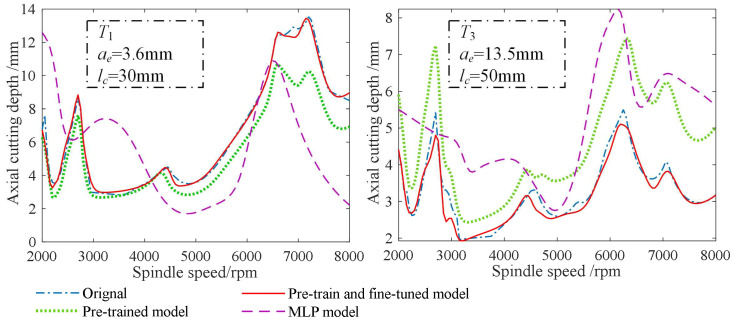
SLDs plotted by different types of prediction models.

**Figure 8:**
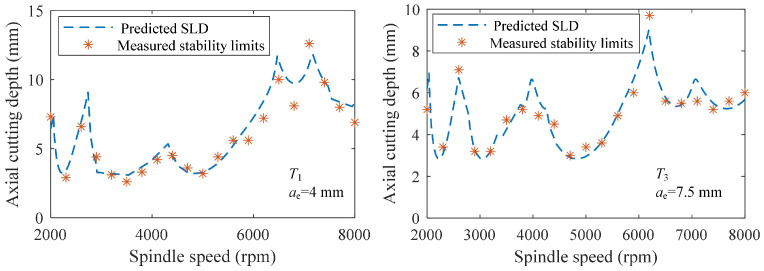
Comparisons between the predicted SLDs and measured stability limits.

For the target tool *T*_1_, the MAPE, RMSE and *R*^2^ values on the 18,150 testing samples were 0.056, 0.892 and 0.987. For the target tool *T*_3_, the MAPE, RMSE and *R*^2^ values on the 18,150 testing samples were 0.062, 0.631 and 0.987. Although the MAPE, RMSE and *R*^2^ values were a little far away from their ideal values of 0, 0 and 1, they still reflected an acceptable prediction accuracy of the pretraining and finetuning-based milling stability prediction model when only four tool overhang lengths and two types of workpiece materials were used to construct the target data. The MAPE, RMSE and *R*^2^ values for the other two models are also shown in Table 7. It can be seen that under the working conditions of the two target tools, the MAPE and RMSE values of the proposed pretraining and finetuning-based model were much smaller than those of the other two models, and the *R*^2^ values were closer to 1 and much higher than those of the two other models. This comparison validates that the proposed method can benefit the building of the stability prediction model when the number of tool overhang lengths and workpiece materials are decreased. In addition, the SLDs plotted using different prediction models are described in Figure 7, where the *a_plim_* values predicted by the proposed method were more consistent with the actual ones calculated through the theoretical milling stability analysis, further indicating the feasibility of the proposed method in training a stability prediction model with fewer tool overhang lengths and workpiece materials. Some experimental stability limits detected from the chatter tests are provided in Figure 8. The curve tendencies of the theoretical SLDs were close to the real conditions, but differences between the predicted and detected stability limits were still observed for some simplifications of the used analytical milling stability model. In the future, we will further extend this proposed method to obtain feedback from few experimental stability limits and improve the prediction accuracy under limited impact tests and chatter experiments.

#### 3.3.2. Influence of the Target Training Data Size

**Figure 9:**
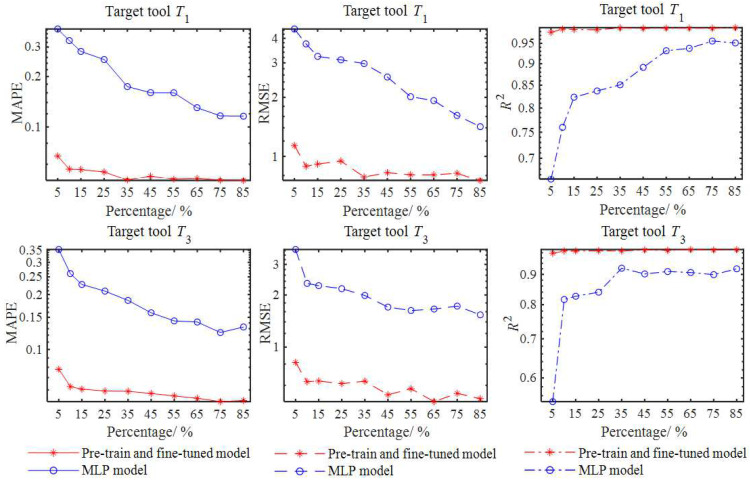
Effects of the target training data size on the prediction models.

In transfer learning, the number of target training samples has a direct impact on the performance of the prediction model [33]. Therefore, different training sample sizes were determined for a comparison and study of their effects on the target milling stability prediction model. The 150 combinations of *a_e_*, *l_c_* and *W_m_* in the source domain were taken as the basis to determine the target training sample sizes. Within the percentage variation range from 5% to 85%, the number of {*a_e_*, *l_c_*, *W_m_*} combinations for the target domain varied from 150 × 0.05 = 8 to 150 × 0.85 = 128. For each target tool, the Latin hypercube sampling method was repeatedly used to sample the combinations of *a_e_*, *l_c_* and *W_m_* within their variation ranges in Table 3. For each combination, 61 pairs of *n* and *a_plim_* were calculated within the range of 2000 rpm to 8000 rpm at an interval of 100 rpm. Therefore, the size of the finally obtained training samples for each target tool varied from 61 × 8 = 488 to 61 × 128 = 7808. Multiple MLPs were constructed through the proposed pretraining and finetuning-based method, and the testing data *D_t_T_*_1*t*_ and *D_t_T_*_3*t*_ obtained in Section 3.1 were still taken to verify the feasibility of the constructed MLPs. The MPAE, RMSE and *R*^2^ values corresponding to different training sample sizes are plotted in Figure 8. The target training samples of *T*_1_ and *T*_3_ were also used to directly train the MLPs for comparisons, and the same testing data *D_t_T_*_1*t*_ and *D_t_T_*_3*t*_ were used to evaluate the corresponding MLPs by calculating the MPAE, RMSE and *R*^2^ values as shown in Figure 9.

It can be seen from Figure 9 that the prediction accuracy of the two types of models was improved with the increase in the target training sample sizes. Within the percentage variation range from 5% to 85%, the pretraining and finetuning-based method effectively improved the prediction accuracy. When the percentage was 5%, the MPAE, RMSE and *R*^2^ values of the transfer learning-based model were already much better than those of the model directly trained using the target training samples, but they still could not meet the needs of practical applications on the whole. When the percentage was increased to 10%, the MAPE, RMSE and *R*^2^ values of the target tools *T*_1_ and *T*_3_ were 0.056 and 0.062, 0.892 and 0.631, and 0.987 and 0.987, respectively, indicating an acceptable accuracy for an actual milling process. When the percentage exceeded 10%, the accuracy of the two types of stability prediction models was further improved, but the advantages of the transfer learning were gradually reduced. Since a larger sample size can significantly increase the cost of data acquisition and labeling, the percentage of 10% was selected in Section 3.1 to construct the target data.

#### 3.3.3. Comparison against Instance-Based Transfer Learning

Currently, instance-based transfer learning has also been widely used in milling stability prediction [34]. The two-stage TrAdaBoost.R2 proposed by Pardoe [35] is commonly used to develop instance-based transfer learning. The basic idea of the two-stage TrAdaBoost.R2 algorithm is that the weighted source data and target data are combined to train a regression model. Compared with the source data, the target data are more important for training the target regression model *f_t_*(·). Therefore, the weights of the target samples are increased iteratively through the adaptive weighting algorithm to emphasize their impact on constructing the regression model. For a milling stability analysis, the instance-based transfer learning requires a basic regression model to predict the limiting axial cutting depth, and the basic regression model should consider the sample weights in the training process. Then, the random forest (RF) and MLP were selected as the basic learners, and 18,150 source training samples and 915 target training samples were combined to train the milling stability regression models using the two-stage TrAdaBoost.R2, respectively. The accuracy of the finally obtained regressors was evaluated using the 18,150 target testing samples. The comparisons between the model-based transfer learning and the instance-based transfer learning are listed in Table 8. The MAPE, RMSE and *R*^2^ values show that the proposed pretraining and finetuning-based method is more suitable for predicting milling stability under multiple machining conditions.

## 4. Conclusions

During the milling process, variation in the tool overhang length, workpiece material or machining parameters leads to a change in milling stability, accordingly. Machine learning can effectively deal with the complex regression problem with a variety of input features. However, as a data-driven method, traditional machine learning requires sufficient training data. Facing multiple milling conditions, many impact tests and milling experiments are needed to obtain sufficient stability data, increasing the time consumption and economic cost. Aiming to reduce the number of impact tests and milling experiments for different tool–workpiece assemblies, this paper proposes a transfer-learning-based milling stability prediction method. First, a source tool was selected to perform impact tests under sufficient tool overhang lengths and conduct milling tests on different workpiece materials. Then, the milling stability analysis was conducted to construct sufficient source stability data. For a new tool, only the representative overhang lengths and workpiece materials are selected for the impact tests and milling experiments to construct the target data. On this basis, a source MLP whose inputs are the machining parameters, tool overhang length and workpiece material is trained on the source data. The structural parameters of the source MLP are retrained on the target data through a global finetuning strategy. With this method, fewer experiments are required to train a tool and workpiece-dependent milling stability prediction model.

Case studies were conducted on a vertical machining center to validate the feasibility of the proposed method. An end mill with a diameter of 12 mm was selected as the source tool, and eight tool overhang lengths and three workpiece materials were determined to carry out the impact tests and milling experiments. The obtained tool tip FRFs and cutting force coefficients were taken to analyze the milling stability and then construct the source stability dataset with 18,150 samples. An accurate source MLP for predicting the *a_plim_* was pretrained using the source data. For the target tools *T*_1_ and *T*_3_ with the diameters of 8 mm and 16 mm, respectively, only four tool overhang lengths and two workpiece materials of each target tool were selected to perform the experiments and construct the target dataset with 915 samples. Then, two target stability prediction models for *T*_1_ and *T*_3_, respectively, were obtained by finetuning the pretrained MLP using the corresponding target samples. For each target tool, the smaller MAPE and RMSE values and the *R*^2^ value close to 1 on the 18,150 target testing samples verify the performance of the proposed method. The proposed method was also compared with the instance-based transfer learning method to validate its advantage in constructing a tool- and workpiece-dependent milling stability prediction model with fewer experiments.

In this work, we adopted the analytical milling stability theory to predict the limiting axal cutting depths for chatter avoidance. However, differences between the theoretical stability limits and those detected by the chatter experiments were often observed. Therefore, in our future work, we will extend the proposed method to introduce a few experimental stability limits in the finetuning stage. It is expected that this could allow for the predicted stability limits to be better adapted to the actual milling operations.

## Figures and Tables

**Table 1:** Specific information of the three different tools.

Tool ID	Materials	Tooth Number	Diameter/mm	Overall Length/mm	Overhang Length Range/mm
*T*_1_	Cemented carbide	2	8	65	20–55
*T*_2_	Cemented carbide	4	12	85	35–70
*T*_3_	High speed steel	3	16	101	50–80

**Table 2:** The cutting force coefficients for different tool and workpiece materials.

Material ID	Material	One-Hot Encoding	Cutting Force Coefficients/(N/mm^2^)					
			*T*_1_		*T*_2_		*T*_3_	
			*K_t_*	*K_r_*	*K_t_*	*K_r_*	*K_t_*	*K_t_*
*W_m_*_1_	AL6061	001	774	260	800	300	984	341
*W_m_*_2_	Cast iron	010	1374	580	1420	670	1746	762
*W_m_*_3_	45 steel	100	1913	658	1977	760	2431	864

**Table 3:** Features of multi-milling conditions for different types of tools and workpieces.

Data Type	Tool ID	Tool Type	*l_c_*/mm	*W_m_*	*n*/rpm	*a_e_*/mm
Data for training	*T*_1_	Target	25, 35, 45, 55	*W_m_*_1_, *W_m_*_3_	[2000, 8000]	[2, 8]
	*T*_2_	Source	35, 40, 45, 50, 55, 60, 65, 70	*W_m_*_1_, *W_m_*_2_, *W_m_*_3_	[2000, 8000]	[2, 12]
	*T*_3_	Target	50, 60, 70, 80	*W_m_*_1_, *W_m_*_3_	[2000, 8000]	[2, 16]
Data for testing	*T*_1_	Target	20, 30, 40, 50	*W_m_*_2_	[2000, 8000]	[2, 8]
	*T*_3_	Target	55, 65, 75, 85	*W_m_*_2_	[2000, 8000]	[2, 16]

Note: *l_c_* is the tool overhang length, *W_m_* is the workpiece material, *n* is the spindle speed and *a_e_* is the radial cutting width.

**Table 4:** Structural parameters of the MLP.

Hidden Layers	Solver	Activation
90 × 90 × 90	LBFGS	Tanh

**Table 5:** Evaluation indexes values of different models trained by Ds.

Model	MAPE	RMSE	*R* ^2^
MLP	0.0271	0.331	0.998
DT	0.0357	0.549	0.993
RF	0.0308	0.461	0.995

**Table 6:** Comparisons of the evaluation index values for different types of MLPs and testing datasets.

MLP Type	Training Dataset	Workpiece Material for the Testing Data	MAPE	RMSE	*R* ^2^
Pretrained MLP	*D_s_*	*W_m_*_1_	0.029	0.754	0.990
	*D_s_*	*W_m_*_2_	0.036	0.560	0.985
	*D_s_*	*W_m_*_3_	0.040	0.280	0.992
MLP*__Wm_*_1_	*D_s_*__*Wm*1_	*W_m_*_1_	0.037	0.759	0.990
MLP*__Wm_*_2_	*D_s_*__*Wm*2_	*W_m_*_2_	0.042	0.663	0.979
MLP*__Wm_*_3_	*D_s_*__*Wm*3_	*W_m_*_3_	0.052	0.355	0.987

**Table 7:** Evaluation index values for different types of prediction models.

Model Type	Training Dataset	*T*_1_			*T*_3_		
		MAPE	RMSE	*R* ^2^	MAPE	RMSE	*R* ^2^
Pre-trained model	*D_s_*	0.175	2.255	0.895	0.404	2.708	0.754
Pre-trained and fine-tuned model	*D_s_* + *D_t_*	0.056	0.892	0.987	0.062	0.631	0.987
MLP trained by target data	*D_t_*	0.329	5.382	0.442	0.358	3.310	0.632

Note: *D_s_* is the source data and *D_t_* is the target data.

**Table 8:** Effects of the target training data size on the prediction models.

Method	*T*_1_			*T*_3_		
	MAPE	RMSE	*R* ^2^	MAPE	RMSE	*R* ^2^
Proposed pre-training and finetuning	0.056	0.892	0.987	0.062	0.631	0.987
Two-stage TrAdaBoost.R2 based on RF	0.175	2.679	0.879	0.252	2.175	0.839
Two-stage TrAdaBoost.R2 based on MLP	0.152	1.919	0.938	0.247	2.086	0.853

## Data Availability

Not applicable.

## References

[1] Jeong K., Seong Y., Jeon J., Moon S., Park J. (2022). Chatter Monitoring of Machining Center Using Head Stock Structural Vibration Analyzed with a 1D Convolutional Neural Network. Sensors.

[2] Ikkache K., Chellil A., Lecheb S., Mechakra H. (2022). Dynamic modeling of milling and effect of tool path on machining stability. Int. J. Adv. Manuf. Technol..

[3] Li Z., He D., Xu K., Xie F., Tang K. (2021). Kinematics-based five-axis trochoidal milling process planning for deep and curved three-dimensional slots. J. Manuf. Sci. Eng..

[4] Altintaş Y., Budak E. (1995). Analytical prediction of stability lobes in milling. CIRP Ann..

[5] Altintas Y., Weck M. (2004). Chatter stability of metal cutting and grinding. CIRP Ann..

[6] Lu X., Wang F., Wang H., Wang X., Si L. (2016). Research progress on chatter stability analysis of milling process. J. Vib. Shock..

[7] Postel M., Özsahin O., Altintas Y. (2018). High speed tooltip FRF predictions of arbitrary tool-holder combinations based on operational spindle identification. Int. J. Mach. Tools Manuf..

[8] Zheng Z., Jin X., Sun Y., Zhang Z., Sun H., Li Q. (2020). Prediction of chatter stability for enhanced productivity in parallel orthogonal turn-milling. Int. J. Adv. Manuf. Technol..

[9] Wang D., Löser M., Ihlenfeldt S., Wang X., Liu Z. (2019). Milling stability analysis with considering process damping and mode shapes of in-process thin-walled workpiece. Int. J. Mech. Sci..

[10] Singh K.K., Kulkarni S.S., Kartik V., Singh R. (2018). A free interface component mode synthesis approach for determining the micro-end mill dynamics. J. Micro Nano-Manuf..

[11] Kolar P., Sulitka M., Janota M. (2010). Simulation of dynamic properties of a spindle and tool system coupled with a machine tool frame. Int. J. Adv. Manuf. Technol..

[12] Wang D., Wang X., Liu Z., Gao P., Ji Y., Löser M., Ihlenfeldt S. (2018). Surface location error prediction and stability analysis of micro-milling with variation of tool overhang length. Int. J. Adv. Manuf. Technol..

[13] Chang L., Weiwei X., Lei G. (2020). Identification of milling chatter based on a novel frequency-domain search algorithm. Int. J. Adv. Manuf. Technol..

[14] Merdol S.D., Altintas Y. (2004). Mechanics and dynamics of serrated cylindrical and tapered end mills. J. Manuf. Sci. Eng..

[15] Kuram E. (2019). Overhang length effect during micro-milling of Inconel 718 Superalloy. J. Braz. Soc. Mech. Sci. Eng..

[16] Lacerda H.B., Lima V.T. (2004). Evaluation of cutting forces and prediction of chatter vibrations in milling. J. Braz. Soc. Mech. Sci. Eng..

[17] Yu W., Chen J., Li Y., Zuo Z., Chen D., An Q., Chen M., Wang H. (2021). Comprehensive study on the cutting specific energy and surface roughness of milled in situ Tib2/Al Composites and Al Alloys. Int. J. Adv. Manuf. Technol..

[18] Qiu J., Wu Y., Zhang K. (2017). Analysis of Influence Factors and Law on Milling Chatter Stability. Mach. Tool Hydraul..

[19] Li C., Long Y., Cui J., Zhao X., Zhao D. (2021). Surface roughness prediction method of CNC milling based on multi-source heterogeneous data. China Mech. Eng..

[20] Ranaweera M., Mahmoud Q.H. (2021). Virtual to real-world transfer learning: A systematic review. Electronics.

[21] Unver H.O., Sener B. (2021). A novel transfer learning framework for chatter detection using Convolutional Neural Networks. J. Intell. Manuf..

[22] Liu X., Li Y., Chen G. (2019). Multimode Tool Tip Dynamics Prediction based on Transfer learning. Robot. Comput. -Integr. Manuf..

[23] Deng C., Tang J., Miao J., Zhao Y., Chen X., Lu S. (2022). Efficient stability prediction of milling process with arbitrary tool-holder combinations based on transfer learning. J. Intell. Manuf..

[24] Postel M., Bugdayci B., Wegener K. (2020). Ensemble transfer learning for refining stability predictions in milling using experimental stability states. Int. J. Adv. Manuf. Technol..

[25] Li X., Grandvalet Y., Davoine F. (2020). A baseline regularization scheme for transfer learning with Convolutional Neural Networks. Pattern Recognit..

[26] Wang J., Chen Y., Feng W., Yu H., Huang M., Yang Q. (2020). Transfer learning with dynamic distribution adaptation. ACM Trans. Intell. Syst. Technol..

[27] Zhou K., Liu Z., Qiao Y., Xiang T., Chen C. (2022). Domain Generalization: A Survey. IEEE Trans. Pattern Anal. Mach. Intell..

[28] Oleaga I., Pardo C., Zulaika J.J., Bustillo A. (2018). A machine-learning based solution for chatter prediction in heavy-duty milling machines. Measurement.

[29] Mishra R., Singh B. (2022). Extenuating chatter vibration in milling process using a new ensemble approach. J. Vib. Eng. Technol..

[30] Kulisz M., Zagórski I., Weremczuk A., Rusinek R., Korpysa J. (2021). Analysis and prediction of the impact of technological parameters on cutting force components in rough milling of AZ31 magnesium alloy. Arch. Civ. Mech. Eng..

[31] Wu X., Liu Y., Zhou X., Mou A. (2019). Automatic identification of tool wear based on convolutional neural network in face milling process. Sensors.

[32] Becherer N., Pecarina J., Nykl S., Hopkinson K. (2017). Improving optimization of convolutional neural networks through parameter fine-tuning. Neural Comput. Appl..

[33] Zhuang F., Qi Z., Duan K., Xi D., Zhu Y., Zhu H., Xiong H., He Q. (2020). A Comprehensive Survey on Transfer Learning. Proc. IEEE.

[34] Chen G., Li Y., Liu X. (2019). Pose-dependent tool tip dynamics prediction using transfer learning. Int. J. Mach. Tools Manuf..

[35] Pardoe D., Stone P. Boosting for regression transfer. Proceedings of the Twenty-Seventh International Conference on Machine Learning (ICML 10).

